# MinION Sequencer Demonstrates Strong DNA Sequencing Accuracy and Identity

**DOI:** 10.17912/micropub.biology.002009

**Published:** 2026-03-22

**Authors:** Andrea Gallagher, Pranav Illendula, Rongsun Pu

**Affiliations:** 1 Biology, Kean University, Union, NJ, US

## Abstract

Nanopore sequencing with the MinION device has revolutionized genomic research. This study evaluated the MinION nanopore sequencer for accuracy, reliability, and reusability. Sequencing of λ phage DNA and *E. coli* DNA achieved high accuracy and identity levels of at least 90%. Results were comparable to previous studies, confirming the MinION’s ability to capture genomic sequences larger than 4.6 Mbp. Decreased accuracy and identity with repeated nanopore use were observed, indicating the need for caution when comparing runs from the same flow cell.

**Figure 1. Sequencing results of λ phage and E. coli K-12:**
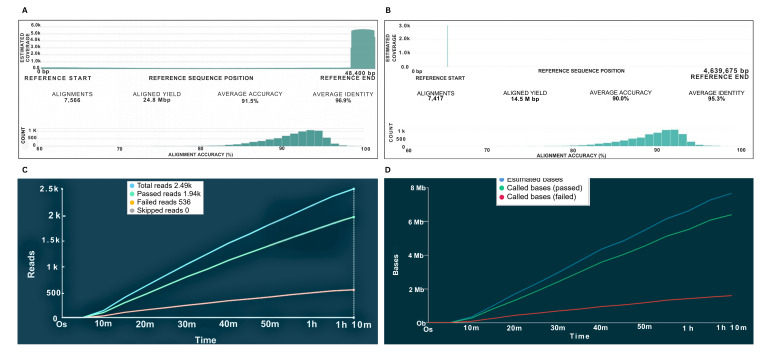
(A) Alignment of λ phage with reference sequence. Reads for the run were 8,015, including 7,566 alignments and 449 reads that could not be aligned. (B) Alignment of *E. coli* K-12 DNA sequence with reference sequence. Reads for the run were 8,015, including 7,417 alignments and 598 reads that could not be mapped. Alignment accuracy is the percentage of all bases in the alignment that are correct, while alignment identity is the percentage of aligned bases that correctly match their corresponding reference bases. Over 7,000 reads were generated in each case and alignment yield in both cases exceeded 10 Mbp. (C) Cumulative reads obtained during sequencing of *E. coli* K-12 genomic DNA. (D) Cumulative bases obtained during sequencing of *E. coli* K-12 genomic DNA. The average sequencing accuracy is 91.5% for λ phage DNA and 90.0% for *E. coli* K-12 DNA, and the corresponding average sequencing identity is 96.9% for λ phage DNA and 95.3% for *E. coli* K-12 DNA. Both λ phage and *E. coli* DNA alignments exhibit lower accuracy than identity, suggesting the presence of significant insertions and/or deletions in the obtained sequences.

## Description

The history of DNA sequencing has been characterized by groundbreaking innovations, with each generation of technology being faster, cheaper, more comprehensive and more convenient. First-generation sequencing technologies culminated in the completion of the draft of the human genome in 2000 (Lander et al., 2001). Second-generation sequencing technologies became widespread in the 2000s, leading to significantly improved sequencing throughput and reduced costs (Mardis 2013; Metzker 2010). Among the various second-generation sequencing technologies available, Illumina has been the dominant short-read sequencing technology with hundreds of different assays for DNA sequencing, RNA sequencing, gene expression studies, and genome assembly (Bentley et al., 2008; Mardis 2017). These assays typically generate sequencing libraries containing short, 200- to 600-bp double-stranded DNA fragments with Illumina sequencing adapters at their ends (Illumina 2023).

The early 2010s saw the advent of third-generation single-molecule sequencing (TGS) that is capable of real-time DNA sequencing. TGS was developed by Pacific Biosciences (PacBio) and Oxford Nanopore Technologies (ONT) (Eid et al., 2009; Jain et al., 2016). The ONT MinION, introduced in 2014, is a compact device designed for rapid DNA or RNA sequencing, fitting in the palm of a hand and operable using a conventional laptop, smartphone, or tablet with the MinIT adapter (Quick et al., 2016; Jain et al., 2018). It works by threading nucleic acid molecules through membrane-bound nanopores, causing nucleotide-specific disruptions in an electric current. This process enables long, continuous reads across 512 nanopore channels, generating up to 30 Gb of data per flow cell. Data analysis can be performed using Nanopore’s freely available software (Lu et al., 2016; Wang et al., 2021).

Despite challenges in base-calling accuracy, ONT sequencing, particularly with the MinION, has proven valuable for rapid clinical diagnoses and research applications due to its real-time capabilities and relatively low cost (Tyson et al., 2018; Wick et al., 2017). This study aims to provide a limited evaluation of the MinION's performance using λ phage DNA and *E. coli* DNA, and offer insights into the reliability of the device's use in genomic sequencing (Loman et al., 2015; Brown et al., 2023).

Our results indicate that the MinION platform achieved 90% or higher accuracy and sequence identity when sequencing genomic fragments up to 4.6 Mb in length, demonstrating that nanopore sequencing with a portable device is capable of producing reliable long-read data. Successful sequencing following DNA library preparation from both the control λ phage and *E. coli* confirmed that the MinION system could be utilized across genomes of different size and complexity. These findings align with previous studies showing that nanopore sequencing is particularly effective for microbial genomes (Judge et al., 2020).

To use the MinION ligation sequencing kit, purified DNA must be obtained. The DNA is then subject to FFPE-repair to correct potential strand damage and ligation to sequencing adaptors, which takes approximately 65 minutes (Castro-Wallace et al., 2017; Oxford Nanopore Technologies, 2023a). Following library preparation, the flow cell must be primed and the sequencing experiment prepared with the ligated DNA, which requires an additional 40 minutes (Tyson et al., 2018; Oxford Nanopore Technologies, 2023b). Proper flow cell priming is critical to ensure optimal pore availability and read quality. Once the sequencing run is initiated in MinKNOW, data acquisition typically proceeds for 6–12 hours (Castro-Wallace et al., 2017). Usable data are generated continuously throughout the run, allowing preliminary analyses to be conducted before sequencing is completed.

Previous research has suggested that MinION sequencing accuracy decreases with shorter read lengths, potentially due to increased base-calling errors and alignment challenges associated with fragmented reads (Lu et al., 2016; Thirunavukarasu et al., 2021). Our findings indicate that MinION sequencing accuracy may be dependent upon sequencing conditions. In the present study, sequencing of λ phage DNA resulted in higher accuracy and sequence identity than sequencing of *E. coli* , consistent with the smaller genome size and reduced complexity of the λ phage. This suggests that factors other than the read length may contribute to observed sequencing performance. One likely contributing factor is the sequencing order and flow cell usage history. The λ phage DNA was sequenced during the first run on the MinION flow cell, whereas *E. coli* DNA was sequenced in a subsequent run. Initial sequencing runs typically benefit from a higher number of active nanopores and more stable pore performance, while later runs may be affected by gradual pore degradation, blockage, or reduced pore availability, potentially leading to lower sequencing accuracy and identity. Although prior studies have reported that nanopores can be reused multiple times with minimal loss of performance (Wick et al., 2017), in our experience, both sequencing accuracy and sequence identity decreased after each successive run on the same flow cell. After four uses, most reads were limited to the kilobase range, and overall read yield was reduced to only a few hundred reads. While these findings are based on a limited number of runs, they indicate that reusing a flow cell may introduce variability that needs to be taken into consideration. Specifically, comparisons between samples sequenced on different runs of the same flow cell may be confounded by pore performance degradation rather than innate biological differences.

The nanopore-based MinION requires minimal manipulation of natural DNA molecules, without relying on sequencing-by-synthesis (Jain et al., 2016). Extremely long reads (over 10 Mbp) and sequence information are obtained directly from DNA efficiently and in real time (Quick et al., 2016). However, a DNA library containing pure DNA must be prepared in order to use the ligation sequencing kit, although there is little requirement for the quantities of nucleic acids (Leggett et al., 2020). Furthermore, Nanodrop can be used to assess the quality of DNA if present at a concentration of > 20 ng/ml (Oxford Nanopore Technologies, 2023c).

The latest base-calling software, guppy5, provides a standard per-base sequencing accuracy of only about 96%, with insertion and deletion errors that are nearly absent in Illumina data (Jain et al., 2018; Tyson et al., 2018). These errors can complicate downstream analyses, including genome assembly, variant detection, and functional annotation. In addition, management of base-calling software can present practical challenges. In order to download the software, one needs to register an account with ONT Community (Oxford Nanopore Technologies, 2023d). Users do not receive notification of software updates and may only realize a new version of the software needs to be downloaded when an error message occurs under "My Device" when running MinKNOW. The current version of EPI2ME Cloud and EPI2ME Agent turned read-only on July 31, 2024, and users must transition to the new EPI2ME Desktop application. This transition necessitates new software installation, validation of system compatibility, and potential restructuring of existing analyses.

A starter pack, including a control cell, a flow cell, λ phage DNA, and some reagents for making DNA libraries, cost $1,000 (Judge et al., 2020). However, this price does not account for additional third-party reagents from New England Biolabs (NEB) required for complete library preparation workflows, including NEBNext FFPE Repair Mix (cat# M6630), NEBNext Quick Ligation Module (cat# E6056), and NEBNext Ultra II End Repair/dA-tailing Module (cat# E7546) (Loman et al., 2015). When these reagents are included, the average cost per run with MinION reagents and required DNA processing kits is typically on the order of $400–$500. More recent published price lists indicate that individual MinION flow cells (“FLO-MIN114” with R10 chemistry) are approximately $800 each, and that control expansion kits (e.g., λ phage DNA) are available for roughly $75 as an add-on consumable from the ONT store (https://store.nanoporetech.com/us/flow-cell-r10-4-1-2025.html). Despite these expenses, the MinION remains significantly more affordable on a per-run basis than many high-throughput sequencing platforms (Adewale, 2020; Zee et al., 2022), especially when factoring in the cost of large instrument needed for high-throughput sequencing.

The relatively low cost, minimal laboratory infrastructure requirements, and ease of operation make the MinION especially suitable for field-based applications and educational settings (Judge et al., 2020). Nanopore sequencing has seen substantial growth in basic and applied research (Hendrix et al., 2021; Leggett et al., 2020; Oehler et al., 2023; Tyler et al., 2018). Its clinical potential is being continuously explored (Chen and He, 2021; Ling et al., 2023; Petersen et al., 2019; Tan et al., 2023). The MinION device is integrated with free, up-to-date bioinformatics tools provided by ONT (Quick et al., 2016). Its portability, affordability, and real-time sequencing capabilities can provide a transformative research and educational experience in DNA sequencing. Our findings also underscore the need for cautious interpretation of sequencing results, particularly in experiments involving multiple sequencing runs per flow cell. Recognizing and accounting for the effects of flow cell reuses will be important for ensuring data reliability and reproducibility in future MinION-based studies.

## Methods

Control λ phage DNA contained within the Ligation Sequencing Kit (SQK-LSK114, ONT) was prepared and sequenced according to the company protocol (Oxford Nanopore Technologies, 2023e). To obtain *E. coli* K-12 DNA, single colonies were obtained from streak plates prepared from the original agar slant. Single colonies were cultured in LB overnight to reach OD _600_ 0.8 and the DNA was extracted by centrifuging 1.5mL of the overnight culture at maximum speed for 1 minute and resuspending the cell pellet in 200µL of TE buffer. Lysozyme (10mg/mL) was added to the cell suspension and the mixture incubated at 37°C for 5 minutes, after which 40µL of 10% SDS and 20µL of 5M NaOH were added and mixed gently by inverting the tube. The tube was incubated for 5 minutes at room temperature, then 150µL of 3M KAc was added and the tube placed on ice for 5 minutes. The tube was centrifuged at maximum speed for 5 minutes and the supernatant transferred to a fresh microcentrifuge tube. An equal volume (about 300 µL) of cold isopropanol was added and mixed gently. The tube was incubated at -20°C for 15 minutes to precipitate the DNA, then the tube was centrifuged at maximum speed for 10 minutes to pellet the DNA. The supernatant was poured off and 500 µL of 70% ethanol was added. The tube was centrifuged at maximum speed for 5 minutes and the ethanol poured off. After air-drying for 10 minutes, the DNA pellet was resuspended in 100µL TE.

DNA sequence was analyzed using MinKNOW software, version 23.04.6 (Oxford Nanopore Technologies, 2023f). NCBI GenBank genome sequence data were used for the alignment of λ phage DNA (National Library of Medicine, 2000) and *E. coli* DNA (National Library of Medicine, 2013). Alignment was analyzed using EPI2ME software, version 5.0.0 (Oxford Nanopore Technologies, 2023g).

## Reagents

**Table:** 

Name	Supplier	Catalog Number
MinION starter pack with flow cell	Oxford Nanopore Technologies	FLO-MIN114
Ligation Sequencing Kit including λ phage DNA	Oxford Nanopore Technologies	SQK-LSK114
NEBNext FFPE Repair Mix	New England Biolabs	M6630
NEBNext Quick Ligation Module	New England Biolabs	E6056
NEBNext Ultra II End Repair/dA-tailing Module	New England Biolabs	E7546
*E. coli* K-12 strain agar slant	Carolina Biological Supply	155065
